# Marginal effects of glucose, insulin and insulin-like growth factor on chemotherapy response in endothelial and colorectal cancer cells

**DOI:** 10.3892/ol.2013.1710

**Published:** 2013-11-27

**Authors:** EKATERINA VOLKOVA, BRIDGET A. ROBINSON, JINNY WILLIS, MARGARET J. CURRIE, GABI U. DACHS

**Affiliations:** 1 Mackenzie Cancer Research Group, Department of Pathology, University of Otago Christchurch, Christchurch 8140, New Zealand; 2 Canterbury Regional Cancer and Blood Service, Canterbury District Health Board, Christchurch 8140, New Zealand; 3 Lipid and Diabetes Research Group, Christchurch Hospital, Christchurch 8140, New Zealand

**Keywords:** proliferation, stroma, low dose, fluorouracil, irinotecan, oxaliplatin

## Abstract

Resistance to chemotherapy is a major clinical issue for patients with colorectal cancer. Obesity has been associated with a poorer outcome and is a possible mechanism of resistance. The aim of the present study was to investigate the effect of obesity-related factors on the cell response to standard chemotherapy in stromal and colorectal cancer cells. Viability was measured following the treatment of colorectal cancer cell lines (WiDr and SW620) and stromal cells (human microvascular endothelial cells) *in vitro* with 5-fluorouracil, irinotecan and oxaliplatin under obesity-related conditions [elevated levels of insulin, insulin-like growth factor-1 (IGF-1) and glucose] and compared with non-elevated conditions. Obesity-related conditions alone increased cell viability and in selected cases, accumulation of the transcription factor, hypoxia-inducible factor-1. However, these conditions did not consistently increase resistance to the chemotherapy agents tested. The combination of IGF-1 and extremely low-dose chemotherapy significantly induced cell viability in WiDr colorectal cancer cells. These *in vitro* results may have clinical importance in an environment of increasing rates of obesity and colorectal cancer, and the frequent under-dosing of obese cancer patients.

## Introduction

Resistance to chemotherapeutic agents, either *de novo* or developing during a course of treatment, is a major clinical issue for patients with colorectal cancer (1–3). Current response rates to combination chemotherapy are ~50%, and as resistance develops in almost all patients, understanding the mechanisms behind this is vital. Despite previous intense investigations, these mechanisms are not completely understood.

For disease stages II and above, chemotherapy is routine, consisting of intravenous 5-fluorouracil (5-FU; or oral capecitabine) with or without oxaliplatin and/or irinotecan (4). 5-FU is an analogue of uracil, which is metabolised intracellularly to toxic compounds, causing DNA damage and the blocking of DNA replication and translation (5). Oxaliplatin is a platinum-based drug, which forms platinum-DNA adducts in cells, causing G_2_ arrest, inhibiting growth and leading to apoptosis (6). Irinotecan, once converted to the active metabolite SN-38, binds to and inhibits topoisomerase I at the initial stages of DNA replication, which leads to cell cycle arrest and DNA damage with subsequent apoptosis (7).

Obesity is an established risk factor for colorectal cancer incidence and mortality (8–10), but the impact on survival and treatment response remains controversial (11–14). In breast cancer patients, the response rate to neoadjuvant chemotherapy (predominantly anthracycline-based regimes) has been lower in overweight and obese patients compared with normal and underweight patients (15). Obesity is associated with insulin resistance, which alters the levels of plasma glucose, insulin and insulin-like growth factor-1 (IGF-1) (16–18).

Insulin is a potent mitogen and stimulates DNA synthesis (19). Experimental models have shown that pretreatment with insulin increases the effect of subsequent 5-FU treatment in the human colon cancer cell line, Ls-174-t (5). Insulin also increases 5-FU uptake and 5-FU-mediated apoptosis. By contrast, insulin has been found to decrease the toxic effects of 5-FU in HT29 colorectal cancer cells (20).

IGF-1 functions as an anti-apoptotic growth factor (21). Breast cancer cells with abnormalities in the IGF-pathway showed IGF-1-mediated suppression of apoptosis and subsequently, were more resistant to doxorubicin and paclitaxel (22). Similarly, IGF-1 increased resistance to 5-FU in the SW480 colon cancer cell line, which was reversible by IGF-1 receptor (IGF-1R) inhibition (23). In addition, HT29 colorectal cancer cells, selected for resistance to 5-FU and oxaliplatin, showed increased expression and activation of IGF-1R (3).

Hypoxic conditions promote the development of treatment resistance, partly through hypoxia-inducible factor-1 (HIF-1)-mediated pathways (24). HIF-1 is the master regulator of molecular responses to hypoxia, controlling >100 genes involved in tumour aggression (25). Previous studies have shown that HIF-1α expression, stability and activity may be modulated by metabolic disturbances, including a number of cytokines and growth factors and specifically, insulin and IGF-1 (26,27).

Conflicting results with regard to the impact of obesity-related factors on chemoresponse have been published previously (5,20,23), as aforementioned. The aim of the current study was to investigate the effect of increased levels of insulin and IGF-1 and altered levels of glucose, on the cellular response to standard chemotherapy *in vitro*. The response of two colorectal cancer cells, one derived from a primary adenocarcinoma (WiDr), the other from a metastatic site of an adenocarcinoma (SW620), was compared with a stromal cell type [human microvascular endothelial cells (HMEC)-1)]. The duration of stimulation (pretreatment time) was also investigated to distinguish between acute and chronic disturbance in the insulin/IGF-1 axis.

## Materials and methods

### Cell culture

Human colon cancer cells (primary adenocarcinoma, WiDr and metastatic adenocarcinoma, SW620; American Type Culture Collection, Manassas, VA, USA) and HMEC-1 cells (Centers for Disease Control and Prevention, Atlanta, GA, USA) were used (28). Cancer cell genotypes are listed in Table I and HMEC-1 cells were assumed to be wild-type (no contrasting evidence was reported). Cells were cultivated in high (25 mM) or normal (5.6 mM) glucose Dulbecco’s modified Eagle’s medium (DMEM; Gibco, Carlsbad, CA, USA) with 10% cosmic calf serum (CCS; Thermo Scientific HyClone, Logan, UT, USA) in standard conditions (humidified at 37°C in 5% CO_2_), unless specified otherwise. Glucose concentrations in cell culture were monitored over time (Optium Xceed; Abbott Diabetes Care, Doncaster, Australia), demonstrating that glucose concentrations reduced by ~18% over 24 h in a confluent cell culture.

### Cell viability assay

Cells were cultivated in DMEM with 10% CCS with high (25 mM) or normal (5.6 mM) glucose concentrations and incubated for 24 h. Plain media, IGF-1 (13 nM; Sigma-Aldrich, St. Louis, MO, USA) or insulin (10 nM; Invitrogen Life Technologies, Carlsbad, CA, USA) were added to the cells 24, 4 or 0 h prior to the addition of 5-FU (0.2–200 μM), oxaliplatin (0.001–100 μM) or irinotecan (0.001–100 μM). Each treatment was tested in four wells per experiment, with three independent experiments, and the cells were treated for 72 h. Cell viability was estimated by standard 3-(4,5-dimethylthiazol-2-yl)-2,5-diphenyltetrazolium bromide (MTT) assay (29), calculated as a percentage of the controls (0 μM of chemotherapy drug) and adjusted for background absorbance. The concentration of drug able to reduce viability to 50% (IC_50_) was calculated from equations obtained by model fitting. Although it is accepted that MTT, an indicator of metabolically active mitochondria, potentially overestimates the number of viable cells compared with several other viability methods (30), it remains widely used in drug discovery and allows for comparisons with previously published data.

### Western blot analysis

Media was replaced with serum-reduced DMEM (0.1% CCS) 24 h prior to and throughout the experiment. The cells were treated for 4 h with IGF-1 (13 nM), insulin (10 nM), CoCl_2_ (100 μM positive control) (31) or plain media (negative control). Nuclear protein fractions were extracted and analysed by western blot analysis following standard protocols (31). A total of 40 μg protein extract was loaded per well for the total and nuclear fractions. Anti-HIF-1α (1:250; clone 54/HIF-1α; BD Biosciences, San Jose, CA, USA) and anti-β-actin (1:2,000; clone AC-15; Sigma-Aldrich) were simultaneously used as primary antibodies to detect HIF-1α and to verify equal loading of protein. Horseradish peroxidase (HRP)-conjugated polyclonal goat anti-mouse antibody (1:1,000; DakoCytomation, Glostrup, Denmark) was used as a secondary antibody. For IGF-1R protein detection, total protein extracts were analysed, using anti-human IGF-1R (1:100; C-20; Santa Cruz Biotechnology, Inc., Santa Cruz, CA, USA) as the primary antibody and HRP-conjugated polyclonal goat anti-rabbit antibody (1:1,000; DakoCytomation) as the secondary antibody.

### HIF-1α cell-based enzyme-linked immunosorbent assay

Cells were plated into 96-well plates provided in the human/mouse total HIF-1α immunoassay kit (R&D Systems, Minneapolis, MN, USA) at recommended concentrations (10^4^/well) and cultivated under standard conditions. The media was replaced with serum-reduced DMEM (0.1% CCS) 24 h prior to and throughout the experiments. The cells were treated with CoCl_2_ (100 μM positive control), plain media (negative control), IGF-1 (13 nM) and insulin (10 nM) for 4 h. The cells were then fixed with 4% formaldehyde and analysed immediately by immunoassay according to the manufacturer’s instructions.

### Data analysis

SPSS 16.0 (SPSS, Inc., Chicago, IL, USA) and Microsoft Excel 2007 (Microsoft Corporation, Redmond, WA, USA) software were used for the statistical analysis and graphical presentation of the results.

For the MTT assay results, several linear regression models using ln transformation of drug concentration (μM) or cell viability (percent) or the two variables together were tested. The model was considered to be a good fit if the R^2^ value was >0.8. Different models were allowed to be used for the various cell lines and chemotherapy drugs. However, within these, the same model was used across the various growth factors, pretreatments and media conditions. Selected models were used to calculate the IC_50_ and ultra-low dose (ULD) values and for multiple regression analysis.

The following linear regression models were selected to fit the viability data according to the R^2^ values: ln transformation of drug concentration for WiDr treated with 5-FU and oxaliplatin, for all treatments of SW620 and for HMEC-1 treated with 5-FU; and ln transformation of cell viability for WiDr treated with irinotecan and for HMEC-1 treated with oxaliplatin and irinotecan. IC_50_ values were calculated from the equations obtained by model fitting. These values were used to compare the effect of growth factors on the cellular response to chemotherapy. Independent sample t-tests were used to compare IC_50_, ULD and HIF-1α protein levels between the various treatments. In the multivariable regression analysis the effect of growth factors on the response to chemotherapy drugs was estimated by B coefficients (regression ‘slopes’).

## Results

### Effect of growth factors and glucose concentrations on cell viability

Glucose concentrations were specifically selected to be clinically relevant and are those used widely in cancer cell culture studies. The lower glucose concentration (5.6 mM) approximates the lower threshold for normal fasting glucose and the high glucose concentration (25 mM) falls in the hyperglycemic range associated with diabetes (32). Specifically, high glucose concentrations are standard in cancer cell culture studies (33). Growth factor concentrations were selected from previously published patient data; 10 nM insulin (plasma, 2 nM) (34) and 13 nM IGF-1 (plasma, 109 ng/ml) (35).

IGF-1 and insulin increased the proportion of cells with metabolically active mitochondria (cell viability) of stromal and cancer cells by between 13 and 55% (HMEC-1 in high glucose with insulin and WiDr in high glucose with IGF-1, respectively). IGF-1 generally increased viability more than insulin (with the exception of WiDr under normal glucose conditions), and an increased viability was more apparent in high glucose than in normal glucose conditions (with the exception of SW620 with IGF-1 and HMEC-1 with insulin) (Table I).

**Figure 1:**
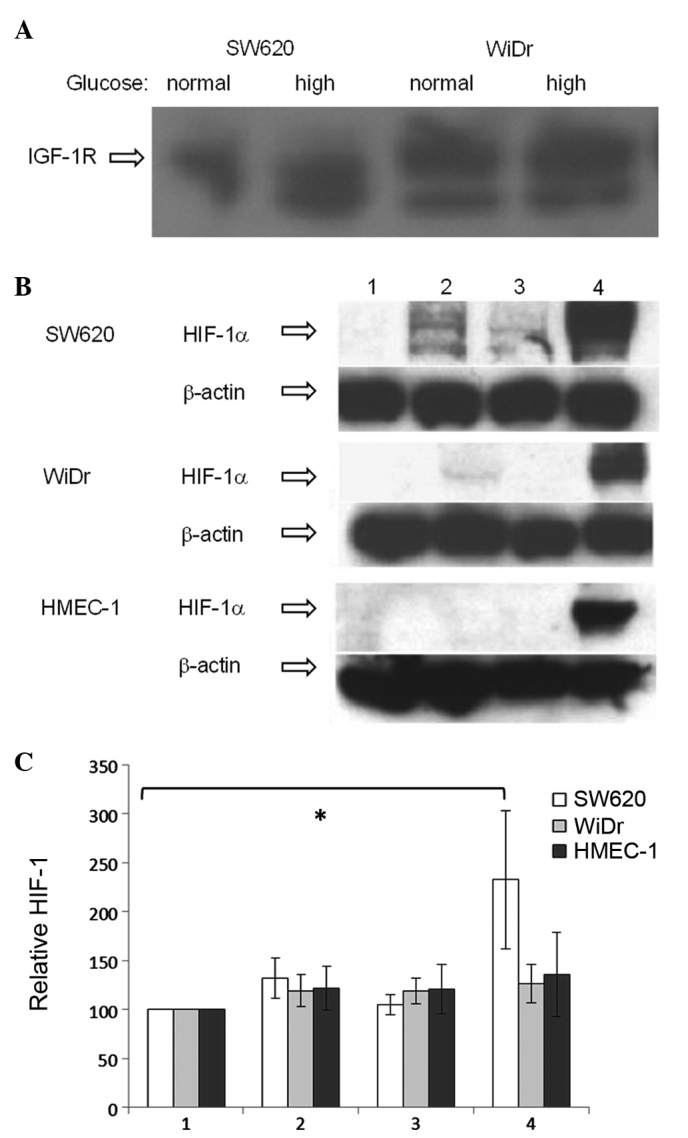
(A) Protein levels of IGF-1R in WiDr and SW620 cells, as detected by western blot analysis. (B) Western blot analysis of HIF-1α protein levels in nuclear fractions of colon cancer cell lines (WiDr and SW620) and human microvascular endothelial cells (HMEC-1) in response to IGF-1 (13 nM), insulin (10 nM) or CoCl_2_ (100 μM). Protein loading, 40 μg/well; HIF-1α band detected at ~120 kDa. Lane 1, untreated (negative control); 2, IGF-1; 3, insulin; and 4, CoCl_2_ (positive control). (C) Cell-based ELISA of HIF-1α protein levels in total protein fraction in response to IGF-1 and insulin. Column 1, untreated (negative control); 2, IGF-1; 3, insulin; and 4, CoCl_2_ (positive control). HIF-1α levels were standardized to untreated cells (100%). *P=0.031. IGF-1R, insulin-like growth factor-1 receptor; HIF-1α, hypoxia-inducible factor-1α; ELISA, enzyme-linked immunosorbent assay; HMEC-1, human microvascular endothelial cells.

Western blot analysis confirmed that the two cancer cell lines expressed IGF-1R (36), with levels not notably affected by glucose concentration (Fig. 1A). IGF-1R levels appeared higher in WiDr compared with SW620, as reported previously (36).

### Effect of IGF-1, insulin and glucose concentrations on cellular response to chemotherapy

The concentrations of chemotherapy agents used in the current study were within the clinically relevant ranges: 5-FU, 0.2–200 μM (maximum plasma concentration, 426 μM); oxaliplatin, 0.001–100 μM (maximum plasma concentration, 3.3 mM); and irinotecan, 0.001–100 μM (maximum plasma concentration, 10 mM) (37–42).

The mean IC_50_ and results of the t-tests for each condition in all cell lines are presented in Table II. For the majority of cell lines, no significant difference was identified in the concentrations of drugs required to reduce IC_50_ between the growth factor-treated and control cells. The duration of incubation with growth factors did not consistently modify the drug response, nor did the glucose concentration. Only one set of data demonstrated significant differences; IGF-1-treated SW620 cells in high glucose were more resistant to irinotecan treatment compared with the controls (P=0.009). Treatment with irinotecan in the presence of insulin under the same conditions showed a similar trend, although a significant difference was not observed (P=0.096).

To compare entire response curves, as opposed to single data points (IC_50_), a multivariable regression model was developed (Table III). As predicted, chemotherapy drug concentration exhibited a significant effect on cell viability in all cases (P<0.001). In the majority of cases, the presence or the duration of pretreatment with growth factors, or the glucose concentration of the media did not significantly change the chemoresponse.

Of the results that showed statistically significant changes, the addition of IGF-1 to tumour cell lines increased the resistance to chemotherapy: WiDr 5-FU in normal glucose at 24 h (P<0.001) and 4 h (P<0.001); WiDr oxaliplatin in normal glucose at 24 h (P<0.001) and 4 h (P<0.001); SW620 5-FU in normal glucose at 0 h (P=0.007); and SW620 irinotecan in high glucose at 4 h (P=0.015).

The addition of insulin to WiDr significantly increased sensitivity to chemotherapy: 5-FU in high glucose at 24 h (P<0.001) and in normal glucose at 0 h (P=0.006); and oxaliplatin in high glucose at 24 h (P=0.021) and in normal glucose at 0 h (P=0.015). In addition, insulin induced variable effects in the SW620 cells, such as increased sensitivity; 5-FU in high glucose at 24 h (P=0.004) and 4 h (P=0.042), and increased resistance; oxaliplatin in high glucose at 0 h (P=0.034) and irinotecan in high glucose at 4 h (P=0.011) and 0 h (P=0.016).

The impact of growth factors in the HMEC-1 endothelial cell line on the chemoresponse was variable; IGF-1 in high glucose marginally increased resistance (oxaliplatin at 24 h, P=0.043), but also sensitivity (5-FU at 4 h, P=0.008; oxaliplatin at 0 h, P=0.011; and irinotecan at 0 h, P=0.008). In normal glucose IGF-1 increased 5-FU sensitivity (0 h, P=0.021), but marginally decreased sensitivity to oxaliplatin (24 h, P=0.038). Insulin increased sensitivity (5-FU in high glucose at 24 h, P=0.01; and in normal glucose at 4 h, P=0.016), but also resistance slightly (oxaliplatin in normal glucose at 0 h, P=0.032).

### Effect of ULDs of chemotherapy on cell viability

WiDr cells showed significantly increased viability when treated with ULDs (defined as 1/1,000 of IC_50_) of chemotherapy in normal glucose conditions with IGF-1, ranging between 182% (oxaliplatin at 4 h, P=0.003) and 240% (5-FU at 4 h, P=0.018), compared with WiDr in normal glucose without growth factors or chemotherapy (viability, 100%) (Table IV). Similar trends were observed at 24 h; WiDr viability in normal glucose with IGF-1 increased to 195% with ULDs of oxaliplatin (P=0.082) and to 283% with ULDs of 5-FU (P=0.088). The viability of cells at ULDs was calculated from the equations obtained by model fitting, and the values were used to compare the effect of growth factors on the cellular response to chemotherapy. No significant differences were identified in ULD response between growth factor-treated and control cells under high glucose conditions or insulin, and this effect was not observed in the SW620 or HMEC-1 cells.

### Effect of IGF-1 and insulin on HIF-1α protein levels

Western blot analysis of the nuclear protein fractions of SW620, WiDr and HMEC-1 showed extremely low or undetectable basal levels of HIF-1α protein (Fig. 1B). As predicted, a marked increase in HIF-1α protein was observed in all cell lines in response to CoCl_2_, an agent used as a positive control as it interferes with HIF-1 degradation (31). An increase in HIF-1α protein levels in response to IGF-1 and insulin treatment was observed in the SW620 cells, with a weaker increase due to IGF-1 and no increase due to insulin in the WiDr cells. No visible changes from the basal HIF-1α protein levels in response to IGF-1 or insulin were observed in HMEC-1.

The effect of IGF-1 and insulin on total HIF-1α protein levels was further quantified using a cell-based immunoassay, with basal levels defined as 100% (Fig. 1C). An increase in HIF-1α protein levels was observed in all 3 cell lines in response to CoCl_2_ [SW620, 233% (P=0.031); WiDr, 126%; and HMEC-1, 136%]. HIF-1α protein levels appeared to be increased in response to IGF-1 in SW620 (132%; P=0.057) and to insulin and IGF-1 in WiDr (insulin, 119%; and IGF-1, 119%) and HMEC-1 (insulin, 121%; and IGF-1, 121%) cell lines, but the increases were not statistically significant.

## Discussion

The present study demonstrated that the obesity-related conditions of elevated glucose, insulin and IGF-1 levels may increase cell viability and in selected cases, resistance to chemotherapy and accumulation of the global transcription factor, HIF-1. The effect became clearer when the total survival pattern of the cells was analysed in a multivariable regression model, instead of analysing single points (IC_50_). Notably, however, a specific induction of cell viability by the combination of obesity-related factors and ULD chemotherapy (0.2 μM 5-FU and 0.04 μM oxaliplatin) was identified. This observation deserves further investigation, since the plasma levels of 5-FU in patients with colorectal cancer stay at 0.01–1 μM for several days following bolus administration (37). Similarly, platinum concentrations stay at >3 μM (1/1,000 of its maximum plasma concentration) for over 500 h following oxaliplatin infusion (38). In addition, extremely low doses of chemotherapy are more likely to circulate in obese cancer patients where under-dosing or capped dosing is common (43). The under-dosing of obese colorectal cancer patients has been shown to result in reduced progression-free and overall survival rates (44).

In WiDr, a significant effect of growth factors was observed more often in normal glucose conditions. By contrast, significant effects in SW620 were mainly observed in high glucose conditions, whereas in HMEC-1, the results did not differ according to glucose concentration. These results indicate that different types of colorectal cancer and stromal cells may vary in their dependence on glucose levels and the insulin/IGF axis, particularly when treated with chemotherapy. This may be associated with the particular metabolic pathways each cancer depends on and may be elucidated further using genetic and proteomic studies.

The results of the multivariable regression analysis from the current study are consistent with certain previously published studies, which have shown a chemosensitivity-promoting effect of insulin (5,45,46) and IGF-1 (47,48), although the effects varied with the cell line. Insulin is likely to act via growth promotion (49) and IGF-1 through the inhibition of apoptosis (50), via the phosphatidylinositol 3-kinase/Akt and mitogen-activated protein kinase/p38 signalling pathways (51).

Hypoxia has been shown to increase drug resistance (24), but the results of the present study show that HIF-1 is unlikely to be the main mechanism underlying IGF-1- and insulin-mediated drug response, as increases in HIF-1 levels were not associated with changes in the chemoresponse. However, the present results confirmed those of previous studies, which demonstrated that insulin, IGF-1 and high glucose levels regulate HIF-1α (27,52).

The present study showed only a marginal impact of the prevailing glucose and insulin/IGF-1 environment on the chemotherapy response in colorectal cells *in vitro*, at clinically relevant 5-FU, oxaliplatin and irinotecan concentrations. However, there was evidence of a proliferative effect on WiDr cells at extremely low concentrations of 5-FU and oxaliplatin, alone or with IGF-1, as may occur in obesity. These *in vitro* results may have clinical implications in Western societies with increasing rates of obesity and colorectal cancer and the frequent under-dosing of obese cancer patients.

## Figures and Tables

**Table I:** Cellular characteristics and viability of cancer and stromal cells grown in high or normal glucose media, following 5-day treatment with insulin or IGF-1 compared with controls with no pretreatment (equivalent to 100%).

		High glucose (25 mM)		Normal glucose (5.6 mM)	
			
	Mutated oncogenes^a^	Insulin (10 nM)	IGF-1 (13 nM)	Insulin (10 nM)	IGF-1 (13 nM)
WiDr^b^	TP53, PIK3CA and BRAF	142±11	155±12	114±21	115±10
SW620^c^	TP53 and KRAS	124±4	136±7	113±10	137±1
HMEC-1^d^		113±3	136±8	116±1	125±4

a (53,54);

b primary and

c metastatic colorectal adenocarcinomas;

d dermal microvascular endothelial cells.

n=9; data are presented as the mean ± SD, according to viability assay. IGF-1, insulin-like growth factor-1; HMEC-1, human microvascular endothelial cells.

**Table II:** Comparison of IC_50_ values of various chemotherapy drugs in WiDr, SW620 and HMEC-1 cells.

	High glucose DMEM									Normal glucose DMEM								
		
	5-FU			Oxaliplatin			Irinotecan			5-FU			Oxaliplatin			Irinotecan		
						
Parameter	No	IGF-1	Insulin	No	IGF-1	Insulin	No	IGF-1	Insulin	No	IGF-1	Insulin	No	IGF-1	Insulin	No	IGF-1	Insulin
Pretreatment, 24 h																		
WiDr																		
IC_50_, μM	200.00	146.30	145.40	37.17	9.58	4.94	17.58	11.11	13.99	114.39	200.00	139.41	70.07	77.83	41.09	15.1	19.32	16.70
P-value		0.29	0.18		0.17	0.17		0.42	0.62		0.26	0.78		0.94	0.70		0.60	0.54
SW620																		
IC_50_, μM	19.41	11.12	7.47	0.44	0.39	0.37	1.19	2.32	0.71	41.84	19.25	33.39	1.38	0.51	0.67	2.86	3.38	4.15
P-value		0.21	0.15		0.86	0.86		0.42	0.58		0.45	0.76		0.32	0.40		0.88	0.74
HMEC-1																		
IC_50_, μM	42.48	83.48	17.64	24.84	31.78	20.66	6.61	3.44	3.98	136.25	138.38	133.42	11.35	21.96	14.69	5.41	4.68	3.01
P-value		0.54	0.32		0.27	0.35		0.35	0.44		0.98	0.97		0.46	0.56		0.69	0.20
Pretreatment, 4 h																		
WiDr																		
IC_50_, μM	144.72	131.62	156.61	14.40	3.33	9.14	14.03	10.49	13.47	166.68	194.02	117.84	45.44	25.91	20.51	16.02	16.92	14.60
P-value		0.88	0.87		0.35	0.62		0.40	0.79		0.5	0.42		0.49	0.43		0.85	0.61
SW620																		
IC_50_, μM	51.95	43.07	8.87	0.53	1.04	0.58	0.09	0.69	1.30	74.28	67.20	61.72	12.19	0.59	3.38	67.90	1.12	3.34
P-value		0.86	0.40		0.28	0.92		**0.01**	0.10		0.94	0.88		0.38	0.53		0.42	0.43
HMEC-1																		
IC_50_, μM	28.84	13.46	22.78	21.31	16.84	20.05	3.02	3.26	3.43	139.51	77.25	72.07	3.48	13.42	4.60	2.08	3.91	2.01
P-value		0.16	0.57		0.44	0.79		0.87	0.49		0.51	0.49		0.16	0.80		0.34	0.98
Pretreatment, 0 h																		
WiDr																		
IC_50_, μM	162.58	138.73	139.19	28.56	9.05	7.94	12.08	12.76	9.59	156.15	106.05	156.15	73.15	14.77	11.90	18.42	16.91	11.99
P-value		0.74	0.76		0.54	0.52		0.89	0.63		0.45	0.48		0.46	0.44		0.66	0.11
SW620																		
IC_50_, μM	21.34	39.25	40.56	0.56	0.79	1.32	3.04	26.46	28.90	11.19	37.93	12.56	0.54	0.93	0.55	2.49	5.45	2.10
P-value		0.61	0.56		0.67	0.43		0.41	0.43		0.41	0.84		0.26	0.96		0.48	0.81
HMEC-1																		
IC_50_, μM	29.04	21.79	35.17	20.43	11.50	22.82	4.41	2.34	4.41	151.19	99.31	137.24	6.44	5.48	9.53	2.97	4.72	4.41
P-value		0.43	0.59		0.21	0.70		0.21	1.00		0.52	0.87		0.83	0.59		0.59	0.64

For each experiment, cells pretreated with IGF-1 or insulin were compared with the control with no pretreatment; n≥3, independent experiments/condition. Statistically significant differences (P≤0.05) are highlighted with bold font. 5-FU, 5-fluorouracil; IGF-1, insulin-like growth factor-1; HMEC-1, human microvascular endothelial cells; DMEM, Dulbecco’s modified Eagle’s medium.

**Table III:** Multivariable regression analysis of viability in response to various treatment conditions.

	WiDr						SW620						HMEC-1					
			
	High glucose DMEM			Normal glucose DMEM			High glucose DMEM			Normal glucose DMEM			High glucose DMEM			Normal glucose DMEM		
						
Parameter	Drug	IGF-1	Insulin	Drug	IGF-1	Insulin	Drug	IGF-1	Insulin	Drug	IGF-1	Insulin	Drug	IGF-1	Insulin	Drug	IGF-1	Insulin
5-FU																		
24 h																		
R^2^	0.581			0.617			0.794			0.761			0.753			0.513		
B	−12.22	−12.553	−31.381	−20.081	71.588	8.719	−10.82	−6.325	−12.622	−14.976	−0.433	2.13	−12.578	1.5	−14.571	−8.327	−0.356	−10.786
P-value	**<0.001**	0.144	**<0.001**	**<0.001**	**<0.001**	0.542	**<0.001**	0.135	**0.004**	**<0.001**	0.945	0.734	**<0.001**	0.789	**0.01**	**<0.001**	0.955	0.088
4 h																		
R^2^	0.543			0.796			0.442			0.268			0.841			0.513		
B	−10.45	−7.15	−1.72	−19.332	45.551	7.633	−18.843	7.483	−35.412	−20.218	20.657	16.618	−12.158	−10.881	−4.618	−6.392	−5.099	−12.119
P-value	**<0.001**	0.323	0.811	**<0.001**	**<0.001**	0.341	**<0.001**	0.663	**0.042**	**<0.001**	0.416	0.513	**<0.001**	**0.008**	0.25	**<0.001**	0.299	**0.016**
0 h																		
R^2^	0.488			0.589			0.69			0.799			0.878			0.57		
B	−10.63	15.076	−0.43	−15.501	−19.406	−28.259	−12.532	4.996	11.183	−10.29	10.724	0.818	−11.755	−3.533	0.716	−6.921	−10.993	−5.923
P-value	**<0.001**	0.078	0.959	**<0.001**	0.061	**0.006**	**<0.001**	0.432	0.082	**<0.001**	**0.007**	0.835	**<0.001**	0.284	0.827	**<0.001**	**0.021**	0.207
Oxaliplatin																		
24 h																		
R^2^	0.74			0.586			0.823			0.835			0.953			0.799		
B	−8.65	−4.03	−15.98	−13.796	58.84	12.797	−9.218	−2.779	−7.768	−11.521	−10.129	−6.099	−0.023	0.117	−0.086	−0.025	0.305	−0.1
P-value	**<0.001**	0.552	**0.021**	**<0.001**	**0.001**	0.439	**<0.001**	0.607	0.153	**<0.001**	0.133	0.362	**<0.001**	**0.043**	0.128	**<0.001**	**0.038**	0.489
4 h																		
R^2^	0.851			0.792			0.715			0.536			0.949			0.814		
B	−8.86	−8.043	5.36	−13.144	40.668	9.011	−12.64	12.225	0.835	−15.765	−18.702	4.921	−0.025	−0.087	−0.009	−0.023	0.111	−0.069
P-value	**<0.001**	0.102	0.273	**<0.001**	**<0.001**	0.328	**<0.001**	0.243	0.936	**<0.001**	0.334	0.798	**<0.001**	0.181	0.891	**<0.001**	0.369	0.579
0 h																		
R^2^	0.581			0.704			0.854			0.887			0.905			0.772		
B	−8.57	14.16	−2.5	−10.89	−8.12	−23.3	−10.538	3.281	12.353	−9.431	5.029	0.02	−0.027	−0.262	0.074	−0.031	0.375	0.41
P-value	**<0.001**	0.146	0.769	**<0.001**	0.388	**0.015**	**<0.001**	0.566	**0.034**	**<0.001**	0.253	0.996	**<0.001**	**0.011**	0.465	**<0.001**	0.051	**0.032**
Irinotecan																		
24 h																		
R^2^	0.832			0.793			0.78			0.749			0.843			0.555		
B	−0.043	−0.089	0.074	−0.062	0.315	0.072	−8.417	3.646	−6.583	−8.786	5.782	3.273	−0.069	0.037	0.088	−0.04	0.422	0.362
P-value	**<0.001**	0.69	0.739	**<0.001**	0.115	0.711	**<0.001**	0.52	0.248	**<0.001**	0.383	0.621	**<0.001**	0.881	0.719	**<0.001**	0.181	0.254
4 h																		
R^2^	0.914			0.808			0.707			0.493			0.869			0.681		
B	−0.046	−0.239	−0.066	−0.065	0.083	−0.022	−9.722	20.874	22.045	−12.972	−12.462	−2.064	−0.073	−0.146	0.148	−0.033	0.115	−0.168
P-value	**<0.001**	0.142	0.682	**<0.001**	0.659	0.906	**<0.001**	**0.015**	**0.011**	**<0.001**	0.468	0.904	**<0.001**	0.604	0.601	**<0.001**	0.574	0.415
0 h																		
R^2^	0.885			0.854			0.694			0.838			0.924			0.572		
B	−0.047	0.014	−0.108	−0.052	0.029	−0.297	−8.829	12.802	19.176	−7.507	7.323	0.014	−0.108	−0.337	−0.013	−0.037	0.174	−0.027
P-value	**<0.001**	0.943	0.578	**<0.001**	0.281	0.611	**<0.001**	0.104	**0.016**	**<0.001**	0.097	0.997	**<0.001**	**0.008**	0.913	**<0.001**	0.475	0.915

Statistically significant differences (P≤0.05) are highlighted with bold font. R^2^ indicates level of fit to model and the B coefficient indicates the regression slope (negative, sensitive; positive, resistant). 5-FU, 5-fluorouracil; IGF-1, insulin-like growth factor-1; HMEC-1, human microvascular endothelial cells; DMEM, Dulbecco’s modified Eagle’s medium.

**Table IV:** Comparison of ULD effects of various chemotherapy drugs in WiDr, SW620 and HMEC-1 cells.

	High glucose DMEM									Normal glucose DMEM								
		
	5-FU			Oxaliplatin			Irinotecan			5-FU			Oxaliplatin			Irinotecan		
						
Parameter	No	IGF-1	Insulin	No	IGF-1	Insulin	No	IGF-1	Insulin	No	IGF-1	Insulin	No	IGF-1	Insulin	No	IGF-1	Insulin
Pretreatment 24 h																		
WiDr																		
ULD, % viability	158.17	168.8	125.46	103.76	125.05	105.41	90.36	109.5	89.22	149.58	283.39	176.06	114.75	194.54	131.1	102.72	181.35	117.53
P-value		0.804	0.17		0.271	0.894		0.378	0.927		0.088	0.461		0.082	0.436		0.43	0.414
SW620																		
ULD, % viability	122.42	139.86	111.91	113.38	122.15	102.32	105.83	119.95	98.59	182.65	146.21	164.41	123.79	129.72	135.22	93.65	121.95	116.43
P-value		0.338	0.641		0.251	0.558		0.112	0.658		0.324	0.693		0.704	0.691		0.128	0.341
HMEC-1																		
ULD, % viability	147.85	144.61	117.25	91.98	94.92	78.18	89.53	102.14	71.14	118.89	122.22	107.01	75.01	92.94	72.21	61.48	87.38	71.11
P-value		0.889	0.228		0.795	0.152		0.4	0.136		0.862	0.619		0.448	0.787		0.447	0.724
Pretreatment 4 h																		
WiDr																		
ULD, % viability	127.37	132.37	135.44	102.25	113.3	118.13	91.07	83.81	94.34	144.82	239.52	182.69	104.38	181.73	134.42	107.06	132.64	107.58
P-value		0.83	0.747		0.224	0.17		0.598	0.699		**0.018**	0.232		**0.003**	0.078		0.423	0.972
SW620																		
ULD, % viability	191.21	232.28	117	135.51	164.54	111.75	103.67	144.75	102.83	146.65	221.37	208.63	125.87	189.04	161.85	109.53	170.64	140.75
P-value		0.711	0.378		0.5	0.403		0.226	0.946		0.516	0.561		0.421	0.526		0.432	0.573
HMEC-1																		
ULD, % viability	141.66	131.75	128.54	85.62	74.83	80.42	81.2	69.32	76.69	100.87	106.02	94.57	58.83	68.49	54.94	60.45	63.28	58.62
P-value		0.507	0.311		0.226	0.478		0.872	0.761		0.796	0.784		0.458	0.768		0.861	0.921
Pretreatment 0 h																		
WiDr																		
ULD, % viability	117.1	157.06	123.6	93.93	132.27	101.42	90.68	100.67	78.24	173.63	170.8	145.77	127.95	135.3	116.12	131.87	116.16	91.93
P-value		0.249	0.782		0.299	0.525		0.737	0.551		0.953	0.526		0.731	0.567		0.594	0.203
SW620																		
ULD, % viability	121.4	136.46	151.84	112.97	124.57	73.96	100.79	113.19	119.05	119	127.38	119.01	111.46	121.38	112.61	96.44	108.8	100.36
P-value		0.361	0.199		0.371	0.555		0.107	0.089		0.453	0.999		0.263	0.697		0.101	0.357
HMEC-1																		
ULD, % viability	131.97	136.21	125.41	82.43	71.37	85.95	93.89	72.49	85.04	102.84	105.13	96	68.06	63.88	63.32	65.46	75.61	74.85
P-value		0.646	0.35		0.27	0.56		0.244	0.195		0.938	0.816		0.846	0.812		0.705	0.696

For each experiment, cells pretreated with IGF-1 or insulin were compared with the control with no pre-treatment; n≥3, independent experiments/condition. Statistically significant differences (P≤0.05) are highlighted with bold font. 5-FU, 5-fluorouracil; IGF-1, insulin-like growth factor-1; HMEC-1, human microvascular endothelial cells; ULD, ultra-low dose; DMEM, Dulbecco’s modified Eagle’s medium.
